# Clinical features and risk factors of surgical site infections in HIV-negative patients with cryptococcal meningitis underwent ventriculoperitoneal shunt operations: a retrospective study

**DOI:** 10.1186/s12879-022-07719-2

**Published:** 2022-09-14

**Authors:** Lejia Xu, Jianyun Zhu, Xiaoyun Wang, Guofen Zeng, Zhiliang Gao, Jing Liu

**Affiliations:** 1 Department of Pharmacy, Third Affiliated Hospital of Sun Yat-Sen University, Guangzhou, 510630 China; 2 Department of Infectious Diseases, Third Affiliated Hospital of Sun Yat-Sen University, Guangzhou, 510630 China

**Keywords:** Cryptococcal meningitis, Ventriculoperitoneal shunt, HIV-negative, Surgical site infections, Intracranial hypertension

## Abstract

**Background:**

To investigate the clinical features and risk factors of ventriculoperitoneal shunt (VPS) associated surgical site infections (SSIs) in HIV-negative patients with cryptococcal meningitis (CM).

**Methods:**

We retrospectively reviewed the medical records of HIV-negative patients with CM underwent VPS operation admitted to The Third Affiliated Hospital of Sun Yat-sen University in Southwest China over the past 7 years.

**Results:**

193 patients were included, of whom 25 (12.95%) had SSIs in 6 (median duration, 1–48 days) days after operation. Compared with patients without SSIs, patient with SSIs tended to be shorter preoperative stay. 52% patients in SSIs group and 25% patients in no-SSIs group underwent VPS operations within 3 days after admission (p = 0.017). Although body temperature and infectious indicators slightly elevated postoperative in both groups. The patients with SSIs experienced more fever; more central nervous system symptoms; higher PCT value and lower cerebrospinal fluid (CSF) glucose in contrast to the no-SSIs group. Multivariate regression analysis found a 2.653 fold increase in the risk of infection for every 1 °C increase in postoperative body temperature. Among the 25 patients, 9 patients had positive culture results, three samples reported to be oxacillin resistant coagulase-negative Staphylococci.

**Conclusions:**

SSIs was one of the serious surgical complications after VPS operation. High body temperature, the occurrence of dizziness and headache, low postoperative hemoglobin are risk factors. Postoperative patients with high fever, high PCT and low CSF glucose should be paid more attention to.

## Background

Cryptococcal meningitis (CM) is one of the most common clinical presentations of cryptococcosis [1]. There are about 1 million cases of CM worldwide every year [2, 3], which imposes a heavy burden to healthcare systems worldwide. Previous studies tended to focus on the patients with severe immunodeficiency, especially those with human immunodeficiency virus (HIV) [4, 5]. Recently, CM has been increasingly observed in HIV-negative patients [6].

The main clinical manifestations of CM were fever and intracranial hypertension (ICH), including headache, nausea, and vomiting. Persistent ICH could lead to mental abnormalities, consciousness impairment, hearing loss, and visual loss [7]. Cerebral hernia may occur in severe conditions and leading to death, which is one of the major reasons for death in CM patients [8].

Ventriculoperitoneal shunt (VPS), as a solution of high intracranial pressure, is now also widely used in patients with CM-associated ICH [9]. Several studies conducted to verify the therapeutic effects of VPS in non-HIV CM patients confirmed that the placement of a VPS is helpful in decreasing ICH and fungal overload in non-HIV CM patients [9, 10]. However, one of the serious surgical complications, surgical site infections (SSIs) which has a range of 1–39% incidence [11, 12], would lead to re-implantation and longer duration of hospitalization, increased of hospitalization expense and even death [13–15]. Much efforts have been made to identify the characteristics and risk factors for SSIs of VPS operation, in an aim to help clinicians recognize the occurrence of infection earlier thus improve outcome. But most of these studies included patients with intracranial hypertension secondary to a variety of health conditions [16–18]. To the best of our knowledge, there are rarely studies describing the clinical manifestations, characteristics, and risk factors of VPS associated SSIs in HIV-negative CM patients. The manifestations of postoperative infection are nonspecific, including fever, neck resistance, cerebrospinal fluid (CSF) leukocyte change [18], which could be confused with the central infection symptoms of CM.

Thus, we performed a retrospective analysis of the episodes of VPS associated SSIs in patients with CM among HIV-negative population to describe the clinical characteristics and risk factors for VPS associated SSIs in patients with CM.

## Patients and methods

### Study population

This is a retrospective case control study including all patients diagnosed with CM and had undergone VPS surgery from January 1st 2014 through 31th December 2020 in The Third Affiliated Hospital of Sun Yat-sen University. Excluded from the study were (1) pregnant/lactating women; (2) death due to non-infectious factors within 7 days after operation; (3) other operations were performed at the same time with VPS; (4) HIV positive patients.

All patients were given antifungal medicine and other symptomatic therapy. The protocol was approved by the Ethics Committee of the Third Affiliated Hospital of Sun Yat-sen University.

### Data collection

Hospital charts were reviewed with a standardized case-report form to retrieve demographic, clinical, radiographic, and laboratory data. Dual data entry by different operators was performed, with preprogrammed consistency checks. The following data were collected: demographics (sex, gender, age and history of diabetes); preprocedure/surgery time; the duration and type of antimicrobial prophylaxis use; body temperature, clinical manifestation, blood test [white blood cells (WBC), hemoglobin, platelets, blood albumin (ALB), C-reactive protein (CRP), and procalcitonin (PCT) levels], cerebrospinal fluid (CSF) profiles (CSF white blood cell count, CSF glucose, CSF protein, and cerebrospinal fluid pressure), preoperative and postoperative.

### Definitions

Diagnostic criteria for CM were positive culture with cryptococcus in CSF, or positive for India ink staining of CSF smear [19] or known history of cryptococcosis, with clinical manifestations of intracranial hypertension.

An infection was considered to be related to the VPS surgery if at least 1 of the following 2 criteria was fulfilled (according to modified criteria for nosocomial infections of the Centers for Disease Control and Prevention [CDC]) [20]: (1) culture positive in the CSF or in wound secretion and the pathogen was interpreted as relevant, or (2) fever (temperature > 38.5 °C), positive culture of blood, symptoms that newly emerged or worse of ICH (headache or dizziness, neck stiffness, cranial nerve signs, or irritability) with no other explainable cause; physician initiation of an appropriate antimicrobial therapy for SSIs. The onset of infection was defined by the first positive culture, the initiation of an appropriate anti-infective treatment for the SSIs, or surgery at the site of the shunt (whichever occurred first).

### Statistical analysis

SPSS statistics version 25.0 (IBM, Armonk, New York, USA) was used to analyze the data. Continuous variables were presented as Median ± Quartile Range (QR) for non-normal data, or Mean ± standard deviation (SD) for normal data, and categorical variables were summarized as frequencies. T test was used to compare normally-distributed, while Mann–Whitney U test or Wilcoxon signed rank test were used to compare non-normally distributed data, respectively. Categorical variables were compared using the McNemar test, Chi-squared test or Fisher’s exact test. P values for which smaller than 0.05 were considered statistically significant. The multivariate logistic regression was used to analyze the risk factors for infection after VPS.

## Results

### Demographic characteristics

**Fig. 1:**
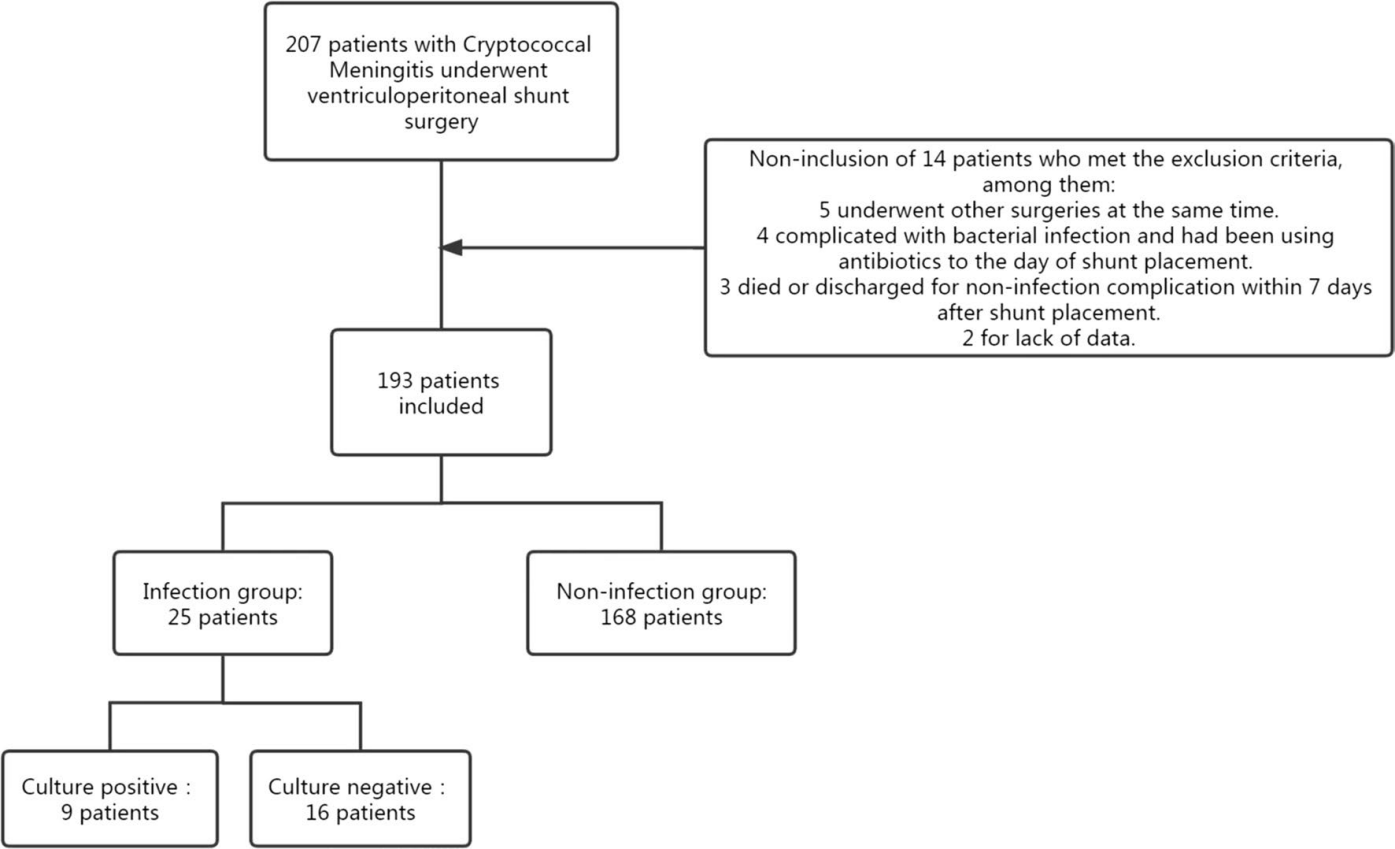
Inclusion and exclusion criteria for cases in this study

A total of 207 CM patients performed on VPS surgery from January 2014 to December 2020 were identified (Fig. 1). Of these, 3 patients died or discharged for non-infection complication within 7 days after the surgery, 5 patients underwent other surgery at the same time, 4 patients diagnosed infection preoperative, and 2 patients lack of data. Therefore, 193 VPS surgery cases were included. According to the infection identification, 25 patients develop surgery-associated infection after operation, the incidence of SSIs associated with VPS operation is approximately 13%. The demographic characteristics of this population were shown in Table 1. 72% of the patients were male. The mean patient age was 45 years (range, 5–72 years). The mean duration of hospital stay was 40 days (range, 4–122 days). 52 patients were admitted to intensive care unit after surgery.

**Table 1:** Demographic data for the 193 CM patients underwent VPS

Variable	Episodes (n = 193)
Male	139 (72.02%)
Age	45.28 ± 15.34
History of diabetes	23 (11.92%)
History of chronic liver disease	33 (16.58%)
History of chronic kidney disease	11 (5.70%)
Length of hospital stay (day)	40.12 ± 15.76
Preoperative duration (day)	10.81 ± 8.20
Duration of surgery (min)	79.28 ± 30.30
Vancomycin impregnated catheters	193 (100%)
Standard prophylactic antimicrobial agents (cefuroxime or cefazolin)	109 (56.47%)
Short duration of prophylactic antibiotics (up to 24 h)	118 (61.14%)

Data were shown as no. (%) or mean ± standard deviation

*CM* cryptococcal meningitis, *VPS* ventriculoperitoneal shunt

### Comparison of clinical symptoms and laboratory indices before and after VPS

The results on preoperative and postoperative changes in clinical symptoms, body temperature, and laboratory characteristics were summarized in Table 2. Body temperature and clinical symptoms were estimated 1 day before and 1 day after the surgery. Intracranial pressure and laboratory characteristics were compared with the last preoperative measurement to the first postoperative measure. After surgery, the body temperature slightly increased, and the percentage of ICH symptoms was slightly decreased while the intracranial pressure decreased approximately 50%. The inflammatory indices, including white blood cell (WBC) of blood, CRP, PCT, elevated to various degrees after surgery. In contrast, there was no significant difference in WBC counts of CSF postoperative. The CSF protein level visibly increased from 0.67 to 1.72 g/L.

**Table 2:** Clinical symptoms, body temperature, and laboratory characteristics changes preoperative and postoperative

Variable	Preoperative	Postoperative	P value
Body temperature (°C)^a^	37.00 ± 0.80	37.30 ± 0.90	< 0.001^***^
Dizziness or headache^b^	159 (81.96%)	142 (73.20%)	0.002^**^
Nausea or vomiting^b^	96 (49.48%)	69 (35.57%)	0.001^**^
Intracranial pressure (mmH_2_O)^a^	326.55 ± 83.96	168.99 ± 44.70	< 0.001^***^
WBC of blood (× 10^9^/L)^c^	8.68 ± 4.44	9.36 ± 5.90	< 0.001^***^
CRP (mg/L)^a^	10.04 ± 26.42	36.52 ± 48.67	< 0.001^***^
PCT (mg/L)^a^	0.09 ± 0.12	0.14 ± 0.25	0.006^**^
WBC of CSF (× 10^6^/L)^a^	60.00 ± 85.00	62.00 ± 96.00	0.630
Albumin of CSF (g/L)^c^	0.67 ± 0.69	1.72 ± 1.57	< 0.001^***^
Glucose of CSF (mmol/L)^c^	1.73 ± 2.11	1.47 ± 1.96	0.009^**^

Data was shown as frequency (relative frequency), mean ± standard deviation or median (quartile range)

*CSF* cerebrospinal fluid, *WBC* white blood cell, *CRP* C-reactive protein, *PCT* procalcitonin

^**^Means that the variables were statistically significant with significance level of 0.01

^***^Means that the variables were statistically significant with significance level of 0.001

^a^Paired samples Wilcoxon signed-rank test, ^b^McNemar test, ^c^Paired samples T test

### Comparison of clinical symptoms and laboratory indices preoperative between infection group and non-infection group

Demographic data, surgery-related information and results of the blood and CSF examination preoperative in infection group and non-infection group were shown in Table 3. There was no significant difference in gender, age and complications between the infection group and non-infection group. Most symptoms, clinical signs and laboratory test results did not differ significantly between the two groups before surgery, except for the preoperative stay. More patients underwent operation within 3 days after admission in the infection group. According to the guiding principles of clinical application of antibiotics in China, cefazolin or cefuroxime is recommended to be prophylactic antibiotics for VPS, and the duration of prophylactic antibiotic should not exceed 24 h. The antibiotic prophylaxis strategy were basically the same in the two group. Standard prophylactic antimicrobial agents (cefuroxime or cefazolin) were used in 56% patient in the infection group and non-infection group, meanwhile 60% patient were given the short duration of prophylactic antibiotics (up to 24 h).

**Table 3:** Demographic data, surgery-related information and results of the blood and CSF examination preoperative in infection group and non-infection group

Variable	SSIs		P value
	No (n = 168)	Yes (n = 25)	
Male^b^	120 (71.43%)	19 (76.00%)	0.635
Age^b^	45.73 ± 15.17	42.28 ± 16.11	0.339
History of diabetes^b^	22 (13.10%)	1 (4.00%)	0.190
History of chronic liver disease^b^	27 (16.07%)	5 (20.00%)	0.622
History of chronic kidney disease^b^	9 (5.36%)	2 (8.00%)	0.595
Preoperative stay < 3 days^b^	42 (25.00%)	13 (52.00%)	0.017^*^
Duration of surgery (min)^c^	79.75 ± 32.01	76.16 ± 14.30	0.583
Standard prophylactic antimicrobial agents (cefuroxime or cefazolin)^b^	95 (56.55%)	14 (56.00%)	1.000
Short duration of prophylactic antibiotics (up to 24 h)^b^	102 (60.71%)	16 (64.00%)	0.829
Preoperative body temperature (°C)^c^	37.04 ± 0.42	37.29 ± 0.52	0.057
Preoperative WBC of blood (× 10^9^/L)^c^	9.06 ± 2.85	10.23 ± 3.03	0.149
Preoperative hemoglobin (g/L)^a^	126.92 ± 17.09	126.84 ± 18.59	0.987
Preoperative CRP (mg/L)^c^	24.55 ± 24.62	21.75 ± 23.89	0.488
Preoperative PCT (ng/mL)^c^	0.20 ± 0.18	0.78 ± 1.04	0.293
Preoperative WBC of CSF (× 10^6^/L)^c^	91.64 ± 70.73	86.83 ± 73.29	0.457
Preoperative protein of CSF (g/L)^c^	0.97 ± 0.6	0.86 ± 0.49	0.549
Preoperative glucose of CSF (mmol/L)^c^	1.97 ± 1.21	1.60 ± 0.97	0.352

Data was shown as frequency (relative frequency), mean ± standard deviation or median (quartile range)

*WBC* white blood cell, *CRP* C-reactive protein, *PCT* procalcitonin, *CSF* cerebrospinal fluid

^*^Means that the variables were statistically significant with significance level of 0.05

^a^Two-samples T test, ^b^Chi-squared test or Fisher’s exact test; ^c^Mann–Whitney U test

### Comparison of clinical symptoms and laboratory indices postoperative between infection group and non-infection group

There were 25 patients (12.95%) who finally developed surgery related infection, with a mean time of 8.88 ± 7.68 days (median time 4 days, range 1–48 days). 72% of the infection (18 persons) developed within 1 week after surgery. The clinical symptoms, body temperature, and laboratory characteristics postoperative in infection group and non-infection group were shown in Table 4. Body temperature would be 0.5 °C higher in the infection group. Intracranial pressure was slightly higher in the infection group, but without statistical significance. There was no significant difference in postoperative blood WBC and CRP between infection group and non-infection group, but the PCT of infection group was slightly higher than that of non-infection group. In the first postoperative cerebrospinal fluid examination, the number of leukocytes in the two groups was equivalent, while the CSF glucose in the infection group was significantly lower than that in the non-infection group. The CSF protein also increased slightly, but with no statistical significance. In the multivariate logistic regression analyses, there were finally two parameters significantly correlated with SSIs (Table 5). High temperature postoperative (OR, 2.653; 95% CI 1.108–6.353), low postoperative hemoglobin (OR, 0.947; 95% CI 0.908–0.988) were risk factors for VPS associated SSIs.

**Table 4:** Clinical symptoms, body temperature, and laboratory characteristics postoperative in infection group and non-infection group

Variable	SSIs		P value
	No ( n = 168)	Yes (n = 25)	
Body temperature (°C)^c^	37.20 ± 0.90	37.70 ± 1.15	0.006^*^
ICH symptoms^b^	123 (79.35%)	21 (87.50%)	0.509
Intracranial pressure (mmH_2_O)^c^	165.99 ± 42.50	187.41 ± 56.93	0.425
Postoperative WBC of blood (× 10^9^/L)^c^	9.34 ± 5.90	9.59 ± 6.56	0.944
Postoperative hemoglobin (g/L)^a^	111.22 ± 17.95	101.12 ± 22.46	0.030^*^
Postoperative CRP (mg/L)^c^	36.50 ± 48.23	36.54 ± 97.69	0.741
Postoperative PCT (ng/mL)^c^	0.13 ± 0.18	0.39 ± 0.39	0.035^*^
Postoperative WBC of CSF (× 10^6^/L)^c^	60.00 ± 98.00	68.00 ± 97.75	0.980
Postoperative protein of CSF (g/L)^c^	1.68 ± 1.51	1.92 ± 1.63	0.079
Postoperative glucose of CSF (mmol/L)^c^	1.57 ± 1.92	0.91 ± 1.74	0.043^*^

Data was shown as frequency (relative frequency) mean ± standard deviation or median (quartile range)

*SSIs* surgical site infections, *ICH* intracranial hypertension, *WBC* white blood cell, *CRP* C-reactive protein, *PCT* procalcitonin, *CSF* cerebrospinal fluid

^*^Means that the variables were statistically significant with significance level of 0.05

^a^Two-samples T test; ^b^Chi-squared test or Fisher’s exact test; ^c^Mann–Whitney U test

**Table 5:** Risk factors for postoperative infection by multivariate logistic regression analysis

Variable	OR	95% CI	P value
Body temperature (°C)	2.653	1.108–6.353	0.028^*^
Postoperative hemoglobin	0.947	0.908–0.988	0.011^*^

*OR* odds ration, *CI* confidence interval

*Means that the variables were statistically significant with significance level of 0.05

Among the 25 patients, 9 patients had positive culture results (Table 6). *Escherichia coli*, *Bacillus circulans* and *Aerococus viridans* were isolated from blood samples of three patients. *Staphylococcus haemolyticus*, *Acinetobacter baumannii*, *Staphylococcus auricularis*, *Streptococcus sanguis* and *Staphylococcus epidermidis* were isolated from cerebrospinal fluid in five patients. One patient was positive for wound secretion culture, which was *Enterobacter cloacae*. All the 25 patients received systemic antibiotic treatment immediately after the diagnosis of infection, median antibiotic treatment duration was 15 days (range, 5–36 days). None of the patients needed surgical procedure of shunt removal or replacement.

**Table 6:** Microbiological findings for episodes of VPS associated SSIs

No	Age/Sex	Pathogen	Specimen collection site
1	55/M	*Staphylococcus haemolyticus* (oxacillin resistance)	CSF
2	15/M	*Staphylococcus auricularis* (oxacillin resistance)	CSF
3	65/F	*Streptococcus sanguis*	CSF
4	42/M	*Staphylococcus epidermidis* (oxacillin resistance)	CSF
5	43/M	*Acinetobacter baumannii* (CRAB)	CSF
6	32/M	*Enterobacter cloacae* (ESBL+)	Wound swab
7	32/M	*Aerococus viridans*	Blood
8	52/M	*Escherichia coli* (ESBL+)	Blood
9	20/M	*Bacillus circulans*	Blood

*M* male, *CSF* cerebrospinal fluid, *ESBL* extended spectyumβ lactamase, *CRAB* carbapenem-resistant *Acinetobacter baumannii*

## Discussion

VPS, which has been proposed to reduce the development of cerebral hernia, may improve the prognosis of CM patients with intolerable ICH. Most of the patients in this study achieving symptom remission significantly after surgery, the intracranial pressure decreased, the ICH symptoms such as dizziness, headache, nausea and vomiting were remarkably responsive to VPS which in common with the previous study [21], suggested that VPS surgery would be efficacy to the CM patients complicated with ICH. However, infection would be one of the major complications of VPS surgery. CSF shunt infections could present with few or no symptoms [18, 22]. Especially in CM patient, the central symptoms of CM which was an infectious disease itself, may interfere with the diagnosis and treatment of CSF shunt infection. Most published studies of CSF shunt-associated infection mainly involved patients with various non-infectious diseases that cause ICH [12, 17, 18, 23]. This study is the first report of SSIs associated with VPS operation in HIV-negative patients with CM.The rate of internalized CSF shunt infection in most studies has ranged from approximately 5% to 15% [17, 18, 22]. Similar to the findings, the infection rate of VPS in HIV-negative patient with CM in our study was 12.95%.

Measures for prevention of SSIs associated with VPS operation include meticulous adherence to surgical and sterile technique (including topical antiseptic), short procedure times, and perioperative antibiotic prophylaxis [24, 25]. Antibiotic-impregnated catheters may be beneficial, too. Use of systemic prophylactic antibiotics decreases rates of CSF shunt infection [26]. According to the guidelines, cefazolin or cefuroxime given 60 min prior to the incision is recommended for patients undergoing clean neurosurgical procedures, including VPS. Cefazolin should be redosed at 4-h intervals until the surgery is over. The IDSA guidelines for the management of healthcare-associated meningitis and ventriculitis suggest that antibiotics be continued for as long as 24 h postoperatively as this is the duration that has been studied [22]. In our study, all the catheters were vancomycin impregnated, and all the patients were given prophylactic antibiotics. But many surgeons hesitated to adhere to the choice of recommended antibiotics agent and the short-course prophylactic antibiotics. Our results demonstrated that application of advanced antibiotics such as ceftriaxone, or prolong the duration, would not reduce infection risk.

Compared with shunt-associated infection in the general population that usually manifests as nonspecific clinical signs and symptoms [18], the clinical manifestations of SSIs associated with VPS in patients with CM seemed to be more obvious, possibly due to the coexistence of CM or unknown immune system disorder in this population. Fever > 38 °C, headache and ICH symptoms were present in over 70% of patients. Although the surgery procedure would lead to a slight increase in body temperature, WBC of blood, CRP, PCT. However, compared with the non-infection group, the postoperative body temperature and PCT of the infection group were much higher, while the CSF-glucose was significantly lower. It suggested that we should consider the possibility of infection when these conditions occur simultaneously after VPS operation in CM patients.

In addition, the majority of VPS associated SSIs occurred within 1 week after surgery (over 60% of the patients). The mean time of infection development after surgery was 8.88 days (range, 1–48 days), which was shorter when compared with the results of other studies that conducted in another population [17, 23]. A possible explanation might be that HIV-negative patient with cryptococcus meningitis may potentially have certain immunodeficiencies that are still undetectable, which therefore makes them more prone to infection and allows the infections manifest more quickly.

This study had limitations due to its retrospective design. Firstly, the diagnosis of infection relied heavily on medical records. If these medical records were not seriously recorded, some infections might be missed. Secondly, missing data was inevitable due to its retrospective design. Thirdly, this study recorded patients’ symptoms and laboratory test results during hospitalization, which partially reflected the short term outcome of HIV-negative patients with CM undergoing VPS operations. Other outcome evaluation methods liked Glasgow outcome score and long-term follow up were needed to accurately access the efficacy of VPS in this population. Nevertheless, we believe that our study is valuable and has several strengths. Firstly, this analysis covered a long period and included a relatively large number of patients. Secondly, the inclusion and exclusion of patients was carried out in strict accordance with the criteria.

## Conclusion

The incidence of VPS associated SSIs in patients with CM was approximately 13%. High body temperature and low postoperative hemoglobin were risk factors for VPS associated SSIs. High postoperative body temperature, low postoperative hemoglobin, high PCT and decreased CSF-glucose might be the indictors for infection. Multivariate logistic regression analyses indicated high temperature postoperative (OR, 2.653; 95% CI 1.108–6.353), low postoperative hemoglobin (OR, 0.947; 95% CI 0.908–0.988) were risk factors for VPS associated SSIs.

## Data Availability

The datasets generated and/or analyzed during the current study are available from the corresponding author on reasonable request.

## Abbreviations

CM: Cryptococcal meningitis
VPS: Ventriculoperitoneal shunt
SSIs: Surgical site infections
HIV: Human immunodeficiency virus
ICH: Intracranial hypertension
WBC: White blood cell
CRP: C-reactive protein
PCT: Procalcitonin
CSF: Cerebrospinal fluid
IDSA: Infectious Diseases Society of America
